# Theoretical and Computational Analysis of a Wurtzite-AlGaN DUV-LED to Mitigate Quantum-Confined Stark Effect with a Zincblende Comparison Considering Mg- and Be-Doping

**DOI:** 10.3390/nano12234347

**Published:** 2022-12-06

**Authors:** Horacio I. Solís-Cisneros, Yaoqiao Hu, Jorge L. Camas-Anzueto, Rubén Grajales-Coutiño, Abdur-Rehman Anwar, Rubén Martínez-Revuelta, Héctor R. Hernández-de-León, Carlos A. Hernández-Gutiérrez

**Affiliations:** 1 Optomechatronics Group, Tecnológico Nacional de México Campus Tuxtla Gutiérrez, Carretera Panamericana Km 1080, Tuxtla Gutiérrez 29050, Mexico; 2 Department of Materials Science and Engineering, The University of Texas at Dallas, Richardson, TX 75080, USA; 3 Laboratory of Nitride Semiconductor Physics, Institute of High-Pressure Physics, Polish Academy of Sciences, Sokolowska 29/37, 01-142 Warsaw, Poland

**Keywords:** simulation analysis, ultraviolet light-emitting diode, AlGaN, quantum-confined Stark effect, p-type analysis

## Abstract

In this work, an AlGaN-based Deep-Ultraviolet Light-Emitting Diode structure has been designed and simulated for the zincblende and wurtzite approaches, where the polarization effect is included. DFT analysis was performed to determine the band gap direct-to-indirect cross-point limit, AlN carrier mobility, and activation energies for p-type dopants. The multiple quantum wells analysis describes the emission in the deep-ultraviolet range without exceeding the direct-to-indirect bandgap cross-point limit of around 77% of Al content. Moreover, the quantum-confined Stark effect on wavefunctions overlapping has been studied, where Al-graded quantum wells reduce it. Both zincblende and wurtzite have improved electrical and optical characteristics by including a thin AlGaN with low Al content. Mg and Be acceptor activation energies have been calculated at 260 meV and 380 meV for Be and Mg acceptor energy, respectively. The device series resistance has been decreased by using Be instead of Mg as the p-type dopant from 3 kΩ to 0.7 kΩ.

## 1. Introduction

Nowadays, III-nitride is one of the most important semiconductor families for device development, such as transistors, lasers, photodetectors, and light-emitting diodes (LEDs) [1]. Different approaches have been developed to emit light in the Deep-Ultraviolet range, under 280 nm, in order to obtain high efficiency and substitute low-pressure mercury lamps [2]. A special effort has been made to determine the ultraviolet susceptibility of different viruses such as SARS-CoV-2, MERS-CoV, and Ebola [3,4]. Here, AlGaN-based deep ultraviolet light emitting diodes (DUV-LEDs) are the current approaches for developing germicidal radiation devices [5]. DUV-LEDs have been developed using nitrides [1,6] due to the wide bandgap [7] and different advantages such as low power, small size with higher efficiency, and wavelength tunability [8]. Nanostructures, nitride-based alloys [9], quantum barrier structures such as graded quantum barriers [10], and quantum wells [11] have been studied to emit in the DUV range, improving the efficiency. Recently, also micro-LEDs have been explored [12,13], resulting in the device size reduction by a light extraction improvement [14].

It is remarkable to mention that, despite external quantum efficiency (EQE) remaining under 10% [15,16,17], some works have overcome this limit. This improvement is commonly performed by considering mirror electrodes, p-AlGaN contact layer instead of typical p-GaN, and high reflective photonic crystal on the p-AlGaN contact layer [18,19]. In general, two different crystal phases to grow III-Nitride semiconductors are explored: hexagonal (wurtzite) and cubic (zincblende) phases [7], while for deep-ultraviolet (DUV) LEDs development, several considerations must be taken into account [20]. On the other hand, wurtzite (wz) materials allow for the incorporation of higher Al concentration to emit in the DUV range in comparison with zincblende (zb) alternative due to the cross-point limit of around 70% of Al in zb-AlGaN-based [7,21]. Commonly, the nanostructures on sapphire substrates have been widely studied [22,23]. However, the limitations of the strong piezoelectric fields attributed to hexagonal semiconductors [24] drive to cubic phase as an essential alternative in III-nitride DUV-LED development. The first consideration is to decrease the effects of intense piezoelectric and spontaneous polarization in the hexagonal phase [25] by substituting it with zb-AlGaN ternary alloy.

Furthermore, recently cubic phase has demonstrated the capability to achieve high hole concentration due to its lower activation energy ~100 meV for p-dopant Mg in zb-GaN. [26] If the hole activation energy is reduced, zb-AlGaN improves DUV-LEDs’ efficiency by reducing optical absorptions in the p-AlGaN contact layer [27]. Therefore, in this work, we start our study by analyzing the bandgap cross-point limit in zb-AlGaN, the Mg, and Be doping for Al content under the direct-to-indirect bandgap cross-point limit by Density Functional Theory (DFT) to be employed in a DUV-LED. Then, once simulated, a discussion on the potential to emit in the germicidal range for the zb-AlGaN approach is performed, focused on Be as p-type doping and its effect on LED performance.

## 2. Theory and Calculations

### 2.1. DFT Calculations

**Figure 1:**
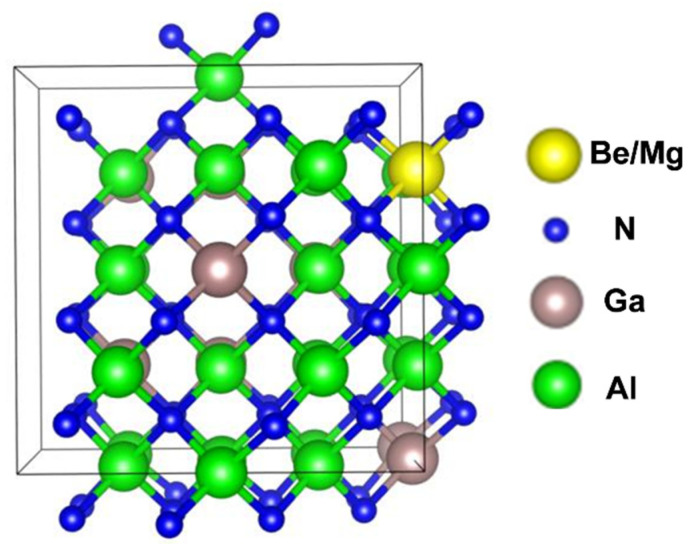
Schematics of the atomic structure of zb-AlGaN with substitute dopant Be/Mg for DFT calculations.

It is critical to focus on the p-doping performance of zb-AlGaN since it is essential to achieving zb-AlGaN-based DUV-LED and other optoelectronic devices. Figure 1 shows the atomic structure of zb-Al_0.7_Ga_0.3_N with p-type substituting dopant (Be or Mg) considered for simulation to obtain the activation energy for the dopants.

**Figure 2:**
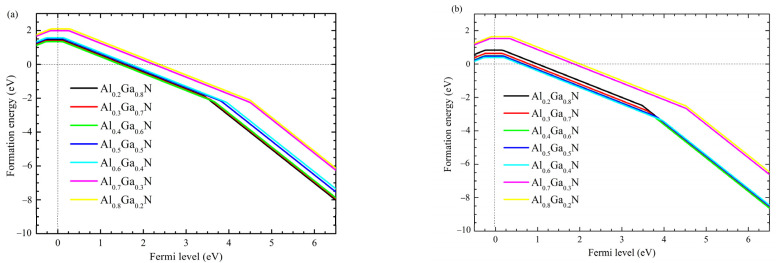
Formation energy of doped AlGaN with (**a**) Be and (**b**) Mg.

The substituting Be and Mg defect formation energy as a function of the Fermi level calculated under N-rich conditions are shown in Figure 2a,b, respectively. Our previous work has shown that in zb-GaN, the Mg acceptor possesses a shallow activation energy level at 100 meV and formation energy of about 1.2 Ev [26].

**Figure 3:**
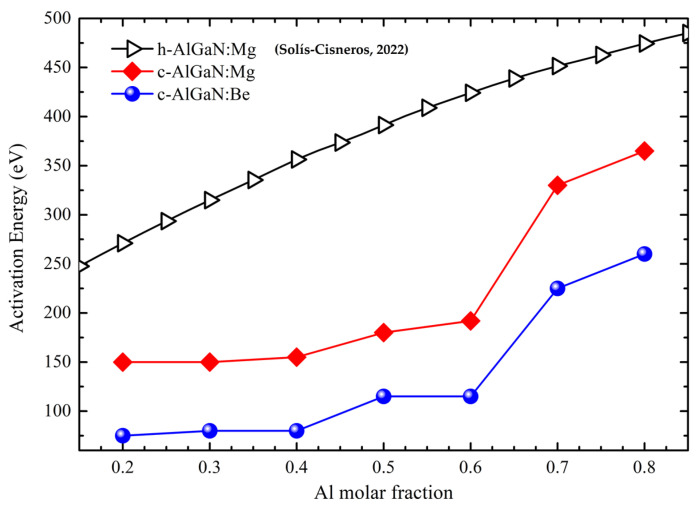
Activation energy comparison between zb- and wz- AlGaN doped with Mg and Be, see ref. [20].

In zb-AlGaN nitride, it is observed that the activation energy for Mg increases with Al content and ranges from 150 meV in zb-Al_0.2_Ga_0.8_N to 370 meV in zb-Al_0.8_Ga_0.2_N, which is lower (Figure 3) compared to the wz-AlGaN:Mg [28].

This behavior shows that Mg remains a shallow acceptor in zb-AlGaN. As a main group-II element, Be is also expected to act as an effective p-type dopant by substituting Al or Ga, though no experiment has proven it until now. Compared to Mg, Be in zb-AlGaN shows higher formation energy but lower activation energy which ranges from 75 meV in zb-Al_0.2_Ga_0.8_N to 260 meV in zb-Al_0.8_Ga_0.2_N. Such shallow activation energy is consistent with previous work [29] and demonstrates that Be is suitable as a p-type dopant in zb-AlGaN. The formation energy of Be as a substitute dopant is higher than Mg due to the larger mismatched atomic radius between Be and Al/Ga. Like Mg, Be in zb-AlGaN also shows an increasing activation energy level with increasing Al content. The effective mass approximation model can explain this. The activation energy is approximated as Coulombic attraction energy between an electron and a nucleus charge similar to a hydrogen atom: [30] (m^*^e^4^)/(32*π*^2^*ε*^2^*ħ*^2^), where m^*^ is the effective mass, *e* is the elementary charge, and *ε* is the dielectric constant. Since GaN has a larger dielectric constant but similar effective mass for holes (compared to AlN), the activation energy of Mg and Be in GaN is smaller and would increase when more Al content is included.

**Figure 4:**
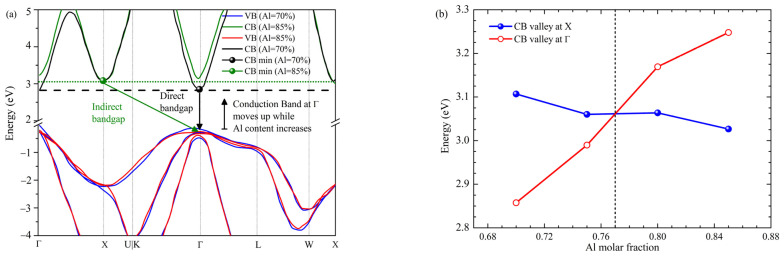
(**a**) Crosspoint interpretation from E-K diagram, (**b**) direct-to-indirect cross-point for zb-AlGaN.

In the context of DUV-LED application, the direct band gap of the active materials should be ~4.8 eV. The zb-GaN has a direct bandgap of 3.2 eV, and zb-AlN shows an indirect bandgap of 5.0–5.3 or even 6.0 eV considering the conduction band edge at Γ-valley for indirect-gap alloys [31]. Thus, incorporating Al into GaN can modulate the bandgap and achieve a 4.8 eV bandgap requirement. However, for alloy zb-AlxGa1-xN, the bandgap could transition from a direct to an indirect gap as x increases. Therefore, the Al content limit is critical information for materials and device design, while AlxGa1-xN maintains a direct bandgap. To answer this question, we have performed a DFT calculation to model the band structures of zb-AlxGa1-xN under various x values, as shown in Figure 4a. Four different Al content levels, 0.7, 0.75, 0.8, 0.85, were studied. It can be seen that zb-Al_0.70_Ga_0.30_N presents a direct bandgap while zb-Al_0.85_Ga_0.15_N exhibits an indirect bandgap due to the conduction band minimum (CBM) shift from Γ point to X point. Note that the actual band gap values are underestimated for zb-AlxGa1-xN, which corresponds to a well-known DFT limitation. Nonetheless, the band features, such as the direct/indirect gap predicted here, are pretty accurate. Further data interpolation (Figure 4b) reveals that the direct–indirect band crossing occurs at x = 0.77 (Al_0.77_Ga_0.23_N), exhibiting a maximum Al content around 0.77, which is valuable guidance to design zb-AlxGa1-xN based DUV range LED and other optoelectronic devices.

### 2.2. LED Structure 

#### Binary Compounds Parameters

Besides the direct-to-indirect cross-point limit, the AlN effective masses and carrier mobilities were calculated by DFT. Binary AlN and GaN values were used to establish a cubic III-nitrides-based alloy simulation set. The zb-LED structure ($P_{{\mathrm{Al}}_{x}{\mathrm{Ga}}_{1-x}N}$) has been approximated by Vegard’s law (Equation (1)) from zb-GaN and zb-AlN parameters shown in Table 1.

(1) $$P_{{\mathrm{Al}}_{x}{\mathrm{Ga}}_{1-x}N}=x\cdot P_{\mathrm{AlN}}+\left(1-x\right)P_{\mathrm{GaN}}-bx\left(1-x\right)$$

where *P* is the parameter to determine, *x* is the Al content in the alloy, and b is the bowing factor. Excluding the Energy gap (E_g_), all bowings factors were not considered, assuming linear dependency on the Al molar fraction. Moreover, the electron affinity has been calculated using the bandgap offset of 0.75 from ΔEc/(ΔEc + ΔEv) [31]. The zb-AlGaN LEDs have been simulated and studied by analyzing the Power Spectral Density and Current-Voltage (I-V) curves obtained from numerical simulation in SILVACO Atlas, meshing the structure and solving using a finite-element approach. Meshing the structure allows exploring graded profiles with a constant approach to defining nodes inside the region with graded composition.

Capture-Scape rates are simulated to present the influence on the active region of the polarization effect. For SILVACO simulation, the Kronig–Penney model for drift-diffusion, Fermi–Dirac for carrier statistics considering incomplete ionization, and the two-band zb model for gain and radiative recombination have been chosen. For zb and wz-approaches, the radiative recombination dependent on polarization (TE, TM) and spontaneous emission rate are modeled using Equation (2).

(2) $$r_{\mathrm{spon}}^{\upsilon }\left(h\nu \right)=\left(\frac{n_{r}e^{2}\omega }{{\pi \mathrm{hc}}^{3}\varepsilon_{0}m_{0}^{2}}\right)\sum_{v}n\rho_{r}^{3D}\left(\hbar \nu \right)f_{c}\left(1-f_{v}\right){\left|M_{b}^{\upsilon }\right|}^{2}$$

where υ is the polarization, n_r_ is the material refractive index, $M_{b}^{\upsilon }$ is a polarization-dependent bulk momentum matrix element, f_c_ and f_v_ are the fermi functions in conduction and valence band, m_0_ is the electron mass in kg, ε_0_ is the vacuum permittivity, and *m_r_* is reduced effective mass obtained from Equation (3).

(3) $$m_{r}={\left(\frac{1}{m_{c}}-\frac{1}{m_{v}}\right)}^{-1}$$

where *m_c_* and *m_v_* are effective masses in the conduction and valence band, respectively, this reduced effective mass is used to calculate ρ_r,_ the density of states given by Equation (4).

(4) $$\rho_{r}^{3D}\left(h\nu \right)=\frac{1}{2\pi }{\left(\frac{2m_{r}}{\hbar^{2}}\right)}^{\frac{3}{2}}\sqrt{h\nu -E_{g}}$$

The scaling factor of polarization is set to zero for the zb approach and 1.0 for wz to add the polarization effect. Moreover, for the strain models for zb and wz, strain tensor calculations are performed in the SILVACO environment by selecting the strained two- and three-band models (zb and wz, respectively) for gain and radiative recombination. Before SILVACO simulation, a finite square well analysis based on effective mass approximation was performed. The first energy level in the quantum well (QW) is calculated by solving the Schrödinger Equation, ensuring wavefunction continuity across the entire quantum well/quantum barrier (QW/QB) heterostructure. Energy is calculated using the Newton–Raphson numerical method to solve Equation (5) [35].

(5) $$\tan\theta =\frac{1}{\theta }\sqrt{\theta_{0}^{2}-\theta^{2}}$$

where θ, and $\theta_{0}$ are described in Equations (6) and (7), respectively. The variable a is the width of the QW in meters, $V_{0}$ is the barrier height in eV, and m is the effective mass in the QW region. This idealized approach has reduced the simulations required in SILVACO to evaluate the structure by narrowing the Al molar fraction and layer thickness ranges.

(6) $$\theta =\frac{a}{2\hbar }\cdot \sqrt{2m\cdot \left(V_{0}-\left|E\right|\right)}$$

(7) $$\theta_{0}=\frac{a}{2\hbar }\sqrt{2m\cdot V_{0}}$$

This quick analysis has been performed to determine a big picture of the thickness and the Al molar fraction in the QW necessary to emit in the DUV range.

**Figure 5:**
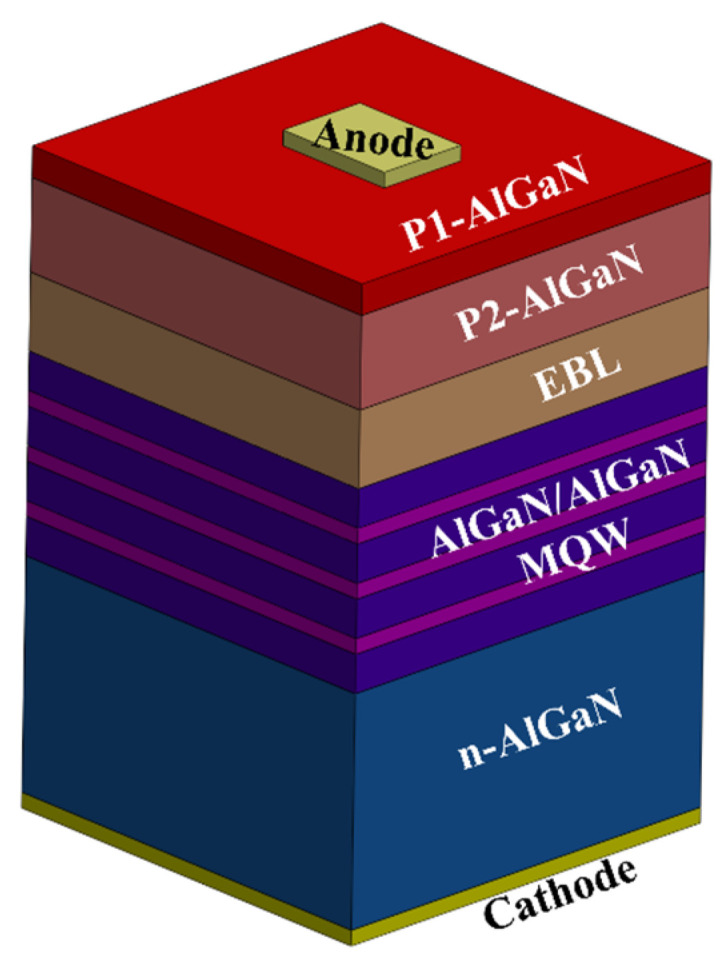
Purposed LED structure.

The structure to simulate using SILVACO Atlas (Figure 5) was determined by combining DFT restrictions on the maximum Al molar fraction in the zb-AlGaN alloy and the QW width effect on the emission. First, the active region is set as an undoped Al_55_Ga_0.42_N/Al_0.75_Ga_0.25_N multiple quantum well (MQW) with three QWs to emit around 275 nm with the zb-AlGaN approach. A thin contact layer (P1) has been implemented to mitigate the low light extraction efficiency due to the high transverse-magnetic polarized light dominance in high Al-rich AlGaN-based MQW structures [36]. The hole injection layer (P2) has been proposed as a 30 nm p-Al_0.50_Ga_0.50_N with p = 2 × 10^19^ cm^−3^. The electron blocking layer (EBL) was considered with p = 2 × 10^19^ cm^−3^ [10,37], lower than the GaN:Mg saturation limit [26,38], and an Al molar fraction of 0.75. For comparison to typical hole concentration, simulations with 5 × 10^18^ cm^−3^ [39] were also performed. The EBL has been explored under the Al molar fraction, avoiding the direct-to-indirect cross-point limit determined by DFT calculations. Finally, the N-layer consists of 500 nm n-Al_0.70_Ga_0.50_N with electron concentration n = 2 × 10^19^ cm^−3^ to broaden the electric field along the active region.

## 3. Results and Discussion

From the data in Figure 1**,** it can be seen that there is a remarkable reduction in activation energy in the p-layer by doping with Be instead of Mg. DFT calculations for zb Mg doping simulation presented an activation energy of 340 meV for p-Al_0.70_Ga_0.30_N, whereas the Be doping shows a lower activation energy of around 200 meV. As shown in Figure 4b, from DFT calculations, the direct bandgap cross-point limit lies at around 0.77 of the Al molar fraction into the AlGaN alloy, consistent with previously reported first-principles [7] and in good agreement with hybrid functional DFT calculations [21]. It would be informative to shed light on the fundamental direct–indirect bandgap transition in AlGaN. For AlGaN, regardless of the Al content, its valence band maximum always stands at Γ point. On the other hand, the conduction band minimum can be either at X or Γ point, depending on their relative energy positions. As Al content increases, the band energy at X gradually decreases, whereas the band energy at Γ gradually increases. This is consistent with the directness feature in AlGaN alloy: at lower Al content, CBM is located at Γ point, so the bandgap is direct; at higher Al content, CBM is at X point, and the bandgap is indirect. The phenomenon that AlN tends to form an indirect gap while GaN tends to have a direct bandgap can be explained by the *s*-*d* and *p*-*d* orbital interaction [40]. AlN has no *d*-orbital in Al, so AlN shows an indirect bandgap, similar to Silicon. In GaN, due to the occupied *d*-orbital in Ga, the *s*-*d* and *p*-*d* orbital couplings push the conduction band energy at X valley up but leave the Γ valley intact. The calculated direct bandgap cross-point limit provides essential material information for AlGaN-based device design. 

**Figure 6:**
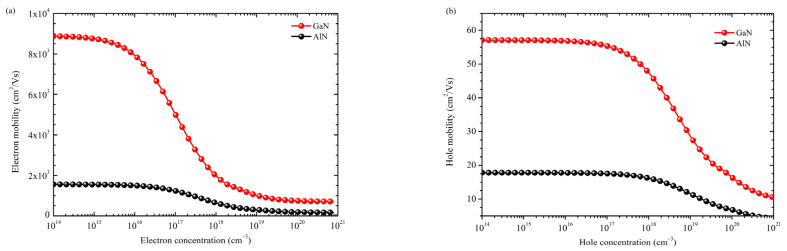
Zb-approach for the carrier mobility as a function of doping concentration (**a**) electron and (**b**) hole in GaN and AlN. The mobility values in GaN and AlN will set the upper the lower limit for carrier mobilities in AlGaN.

In addition to band gap directivity and p-doping capability, carrier mobility is another figure of merit for the AlGaN LED device application. Figure 6 presents the calculated mobilities as a function of doping concentration for both electrons and holes in GaN and AlN. The non-doping limit mobility is limited by phonon scattering [26]. As the doping concentration increases, the Coulombic scattering from ionized dopants increases, so carrier mobility decreases. Phonon scattering and ionized impurity Coulombic scattering are the two fundamental carrier scattering factors in bulk films [41]. It can be seen that GaN shows both high electron and hole mobilities across all the doping concentrations due to the higher intrinsic bulk mobility under the non-doping limit. The mobility values of GaN and AlN will set the upper and lower limit for AlGaN, and depending on Al content, mobilities in AlGaN can be either close to AlN or GaN. 

**Figure 7:**
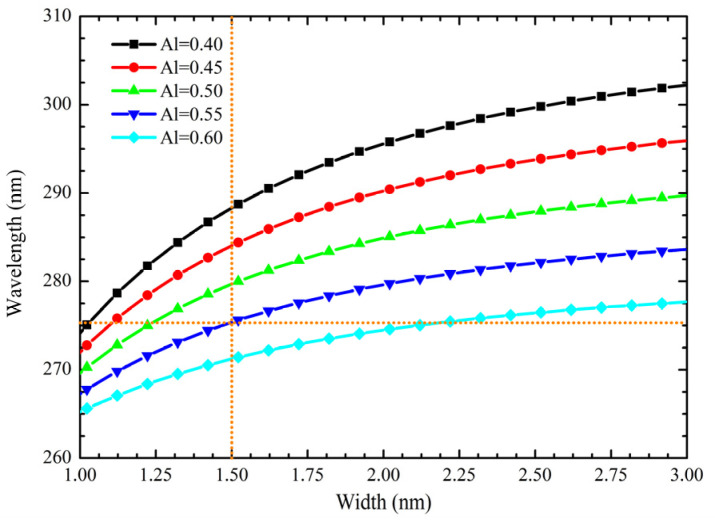
QW width effect on wavelength of zb-AlGaN-based DUV emission using a single quantum well (SQW) active region.

**Figure 8:**
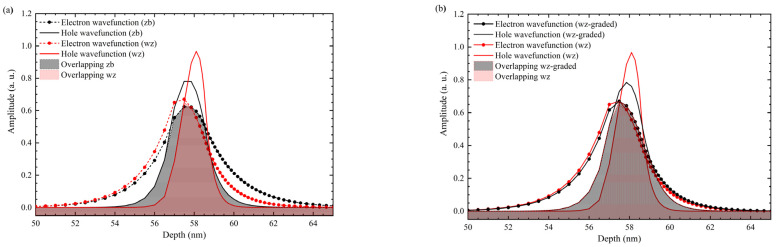
Overlap of the electron-hole wavefunctions in wz- vs. (**a**) zb-, and (**b**) wz-structure with graded QW.

The finite square well calculations are shown in Figure 7, indicating the wavelength emissions as a function of QW width for zb-AlGaN-based DUV-LED. It is important to mention that the calculated difference between the analytical finite square well approach and Silvaco numerical simulations lies under 0.1%. So, finite square QW, with the effective mass approach, is quite accurate in predicting the DUV emission range. The zb-AlGaN ideal approximation shows clear access to the DUV range despite the restriction due to the direct-to-indirect cross-point limit. Although zb-QW reaches emission appropriate for surface disinfection, to achieve shorter wavelengths, the QWs thickness requires thinner than the wz-approach. On the other hand, due to intense piezoelectric polarization in the hexagonal crystals, the probability of overlapping electron-hole wavefunctions is reduced, as shown in Figure 8. Overlapping for wz-approach is calculated from the area under the curve of electron and hole wavefunctions as Equation (8). However, in the absence of band distortions, this overlapping has been increased 58. 46% by using the zb approach compared to the wz-structure, and 59.30% using a wz structure with graded QW instead a wz-structure with constant Al molar fraction.

(8) $$\int_{0}^{L}\min\left(\Psi_{electron},\Psi_{hole}\right)dL$$

where *L* is the thickness to integrate, and the min function denotes de overlapping for electron and hole wavefunctions. As the QW thickness increases, the QW emission wavelength also increases due to the thickness dependence of the quantum-confined Stark effect (QCSE). As it is well known, if an electric field is induced in a confined region such as QW, this QCSE provokes a shift in the wavefunctions (electron to the left and holes to de right), reducing the overlapping. This effect has been compensated by grading Al content in each QW (wz- structure with graded QWs) from 0.55 to 0.60 Al molar fraction with a thickness of 1.5 nm [37].

**Figure 9:**
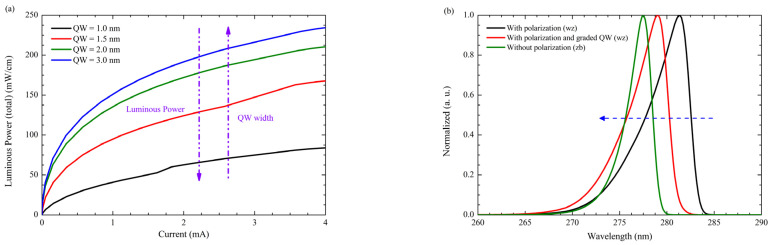
(**a**) QCSE in the wz-approach (**b**) Electroluminescence shift due to polarization in the LED structure as a result of the QCSE.

As shown in Figure 9a. the QCSE in the Luminous Power as a function of the QW thickness could be appreciated. Figure 9b presents the electroluminescence shift due to the polarization effect in the QW region, which is mitigated by the zincblende approach. Parameters for simulation are identical in the three simulations neglecting the electric field due to piezoelectric polarization in the zb-approach.

**Figure 10:**
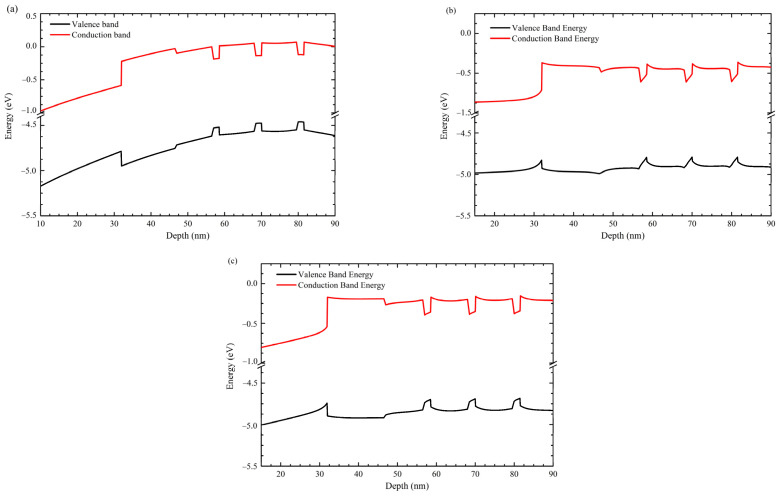
Band diagrams with 5 V forward bias of (**a**) zb-, (**b**) wz-, and (**c**) wz-structure with graded QWs.

Figure 10 presents the band diagrams considering the polarization effect. While zb geometry does not show a piezoelectric band distortion, the wz band distortion could be attenuated by grading the Al content in QWs. On the other hand, p-type region presents a slope in the zb approach which is presumably related to the piezoelectric polarization neglected in the model to emulate the zb-approach.

**Figure 11:**
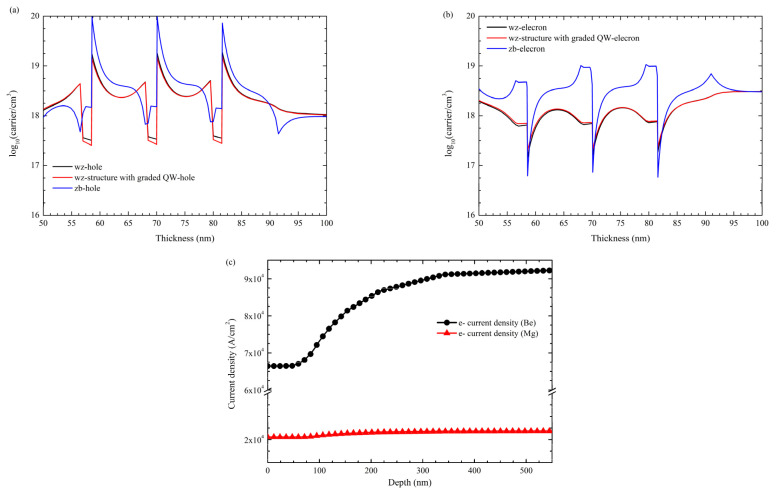
(**a**) Electron concentration for zb-, wz-, and wz-structure with graded QW. (**b**) Hole concentration for zb-, wz-, and wz-structure with graded QW, and (**c**) electron current density for zb-structure with different p-type dopants.

Figure 11a,b shows the carrier concentration due to the polarization effect in three approaches, zb, wz-, and wz- structure with graded QWs structures. By grading the Al content in the QW, the capture electrons concentration diminishes. However, the performance is closer to the zb approach, where electron-hole wavefunctions overlapping increases the direct recombination. Only Mg- and Si-doped P- and N-type layers have been considered, respectively. Nevertheless, current density is improved by substituting Mg with a Be dopant (Figure 11c). 

**Figure 12:**
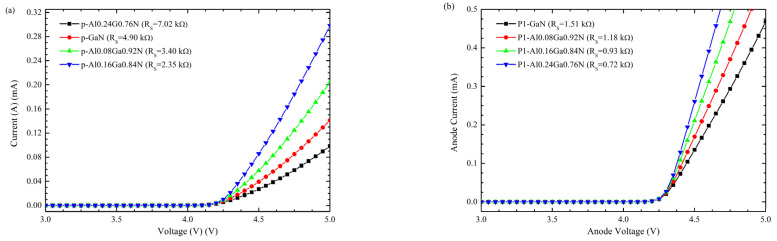
Effect of P1 contact layer incorporation on R_S_ in the zb-approach using (**a**) Mg-dopant and (**b**) Be-dopant.

Additionally, to enhance the electrical characteristics of the DUV-LED, the content of Al in the P1 layer is varied and compared to the structure without the P1 layer and a p-GaN thin contact layer. I-V curves for these simulations are presented in Figure 12, considering incomplete ionization and acceptor activation energy (Figure 2 in Section 2) for Be- and Mg-doping at 260 meV and 370 meV, respectively.

**Figure 13:**
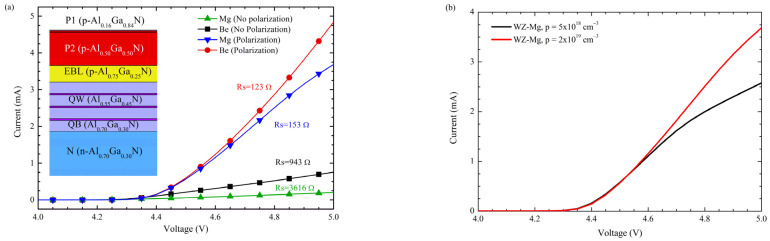
(**a**) Rs comparison between the zb-approach (No polarization) and wz-approach (Polarization) for Be and Mg dopants (**b**) comparison between 5 × 10^18^ cm^−3^ and 2 × 10^19^ cm^−3^.

By selecting Be instead of Mg as a p-type dopant, the Rs of the structure is reduced approximately three times. Figure 13a shows a resistance reduction with the same LED structure and carrier concentrations considering the Mg and Be dopants. Since the p = 2 × 10^−19^ cm^−3^ is a technological challenge in Al-rich layers, it is important to mention that epitaxial growth over the p = 2 × 10^19^ cm^−3^ has been explored experimentally for the zb-GaN [26] and Mg saturation limit was determined in previous work which points to encourage the experimental research on zb-AlGaN doping for DUV applications.

## 4. Conclusions

The simulations show that zb-AlGaN is a candidate for the development of DUV-LED. As presented in this work, a simple approximation, such as SQW, shows that the range of luminescence lies under 275 nm if the QW thickness stands under 1.5 nm and the Al content is limited by the direct-to-indirect cross-point limit of around 77%. Moreover, highly-doped cubic III-nitrides are possible due to their lower activation energy for dopants. The activation energy presented more than 100 meV reduction from 260 to 370 meV using Be instead of Mg. The presented electrical and optical characteristics have been improved for the zb-AlGaN-based LED structure by modifying the p-region modification using a thin AlGaN-based contact layer, where Be, as the p-type dopant, exhibits an LED performance enhancement. A reduction of the Rs from 2.35 kΩ to 0.72 kΩ without the polarization effect in the structure has been determined by considering Be-dopant instead of Mg, presenting Be as an alternative to improve DUV-LED performance by modifying the p-type layers. Moreover, by grading the QW, the polarization effect on bands has been mitigated, enabling future research.

## Figures and Tables

**Table 1:** Parameters to obtain ternary zb-AlGaN.

Parameter	GaN	AlN
Bandgap	3.20–3.30 eV [31]	5.3–6.0 eV [31]
Electron affinity	3.92 eV [31]	2.00 eV [31]
Electron mobility	<1000 cm^2^/(Vs) $\left(n\geq 1\times 10^{19}{\mathrm{cm}}^{-3}\right)$ [This work]	<300 cm^2^/Vs $\left(n\geq 1\times 10^{19}{\mathrm{cm}}^{-3}\right)\;$ [This work]
Hole mobility	<28 cm^2^/(Vs) $\left(p\geq 1\times 10^{19}{\mathrm{cm}}^{-3}\right)$ [This work]	<12 cm^2^/Vs $\left(p\geq 1\times 10^{19}{\mathrm{cm}}^{-3}\right)$ [This work]
Dielectric constant (Static)	10.97 [32,33]	8.90 [33,34]
Effective mass in CB	0.13 [29]	0.84 [This work]
Effective mass in VB	1.40 [29]	2.46 [This work]

## Data Availability

The data presented in this study are available on request from the corresponding author.
